# Images Versus Videos in Contrast-Enhanced Ultrasound for Computer-Aided Diagnosis

**DOI:** 10.3390/s25196247

**Published:** 2025-10-09

**Authors:** Marina Adriana Mercioni, Cătălin Daniel Căleanu, Mihai-Eronim-Octavian Ursan

**Affiliations:** 1 Faculty of Electronics, Telecommunications and Information Technologies, Politehnica University Timisoara, Vasile Parvan Street, No. 2, 300223 Timisoara, Romania; mihai.ursan@upt.ro; 2 Faculty of General Medicine, Victor Babes University of Medicine and Pharmacy Timisoara, Eftimie Murgu Square 2, 300041 Timisoara, Romania

**Keywords:** transformer neural network, computer aided diagnosis, CEUS, contrast-enhanced ultrasound, focal liver lesion, FLL

## Abstract

The background of the article refers to the diagnosis of focal liver lesions (FLLs) through contrast-enhanced ultrasound (CEUS) based on the integration of spatial and temporal information. Traditional computer-aided diagnosis (CAD) systems predominantly rely on static images, which limits the characterization of lesion dynamics. This study aims to assess the effectiveness of Transformer-based architectures in enhancing CAD performance within the realm of liver pathology. The methodology involved a systematic comparison of deep learning models for the analysis of CEUS images and videos. For image-based classification, a Hybrid Transformer Neural Network (HTNN) was employed. It combines Vision Transformer (ViT) modules with lightweight convolutional features. For video-based tasks, we evaluated a custom spatio-temporal Convolutional Neural Network (CNN), a CNN with Long Short-Term Memory (LSTM), and a Video Vision Transformer (ViViT). The experimental results show that the HTNN achieved an outstanding accuracy of 97.77% in classifying various types of FLLs, although it required manual selection of the region of interest (ROI). The video-based models produced accuracies of 83%, 88%, and 88%, respectively, without the need for ROI selection. In conclusion, the findings indicate that Transformer-based models exhibit high accuracy in CEUS-based liver diagnosis. This study highlights the potential of attention mechanisms to identify subtle inter-class differences, thereby reducing the reliance on manual intervention.

## 1. Introduction

An imaging technique that provides real-time, non-invasive, and reasonably priced visualization of vascular structure and tissue perfusion is Contrast-Enhanced Ultrasound (CEUS).

Ultrasound has demonstrated broad utility across various fields of medicine, including hepatology [1], cardiology [2,3,4], gynecology [5,6], and dermatology [7], illustrating its versatility and motivating the adaptation of deep learning (DL) methods across anatomical and disease contexts.

Clinical results have been greatly improved by the extensive use of ultrasound in the identification of renal anomalies, FLLs, and other organ-specific diseases. However, effective analysis of dynamic contrast patterns over time requires professional expertise, making CEUS image and video interpretation a very difficult task [8], only available to very experienced medical personnel. Computer-Aided Diagnosis (CAD) technologies are now important in improving medical imaging diagnostic accuracy [9]. Developing efficient CAD systems for CEUS remains a difficult task due to a number of factors, including a limited number of available patients, a lack of expertise in data labeling, privacy concerns, and data storage [10,11,12].

The increasing usage of Artificial Intelligence (AI) approaches in medical imaging is a result of the need for reliable, efficient, and easily available diagnostic help. Among them, CAD systems are designed to help physicians automate processes such as differential diagnosis, characterization of lesions, and extraction of characteristics. However, because CEUS videos include nuanced enhancement patterns and temporal dynamics, creating reliable CAD solutions for CEUS is extremely challenging [13]. Moreover, one of the most significant advantages of AI in this industry is its ability to speed up medical image analysis. Traditional image interpretation methods are time-consuming and prone to human error. However, AI can analyze and interpret images considerably faster, drastically reducing the time it takes to diagnose a patient. This quickness is especially important in emergency circumstances [14,15].

Convolutional Neural Networks (CNNs) represent cutting edge technology for many medical imaging applications, and they can learn inductive bias [16]. Today, visual data analysis has been completely transformed by recent developments in the DL domain, especially by the introduction of Transformer-based neural networks. Transformers were first created for natural language processing (NLP) but have now been modified for vision applications. An important advantage over CNNs is the remarkable capacity to represent long-range relationships in data. This led us to the idea that Transformers could also be suitable for identifying the complex spatiotemporal correlation aspects of CEUS exploration by considering simultaneous local and global information [17,18].

The Vision Transformer (ViT) architecture is now used in some medical imaging applications [19,20,21]. The transformer excels in capturing long-range relationships through its self-attention mechanism, demonstrating outstanding results in complicated NLP tasks [22,23]. In identifying minor irregularities in anatomical imaging, the local connection among adjacent pixels is significant, along with long-range correlations [24]. On the other hand, CNN solutions are known for their ability to capture local dependencies. The ViT and CNNs have complementary strengths in the processing of medical images for various applications, such as segmentation, classification, and prediction. Many studies have attempted to merge these two designs to collect global and local visual content, resulting in hybrid concepts [25,26,27,28]. CNNs with spatial convolution filters may learn local characteristics; however, shallow CNNs with fewer layers may fail to grasp the image’s global context due to limited abstraction. The ViT uses self-attention to learn long-range dependencies between image patches, whereas patch-based positional encoding may miss local spatial information and fall short of CNN performance on small datasets. Recent investigations, notably in radiology, have emphasized the difficulty of the ViT in detecting modest results in confined spaces [29,30].

### Motivation and Novelty

Liver cancer is the fourth most common kind of cancer worldwide [31]. Romania possesses the highest incidence rates in the EU for liver and nasopharyngeal malignancies among women [32]. Hepatocellular carcinoma, commonly referred to as HCC, is induced by cirrhosis and hepatitis B or C virus [33].

DL techniques have shown promise in pathology. Because manual tumor identification is prone to errors, liver cancers may be misdiagnosed, which might have deadly consequences. CAD methods, on the other hand, save time and need little human intervention [34]. The aim of this study is to develop a CAD system for automated diagnosis of FLLs from CEUS images and videos.

CEUS has emerged as a reliable imaging technology for assessing FLLs [35,36,37], and to address the challenges of CEUS analysis, this article firstly proposes a Hybrid Transformer Neural Network (HTNN) architecture for image-based CAD implementation. The proposed hybrid architecture achieves a balance between performance and computational economy by merging CNNs for efficient local feature extraction with transformers to capture temporal and spatial relationships. Particular focus is placed on creating a tiny, lightweight neural network architecture that can be implemented in real-world clinical situations, possibly on an embedded device, without requiring significant processing resources. Moreover, this approach addresses the limitations of typical medical/CEUS datasets, namely the small number of available patients, while being able to categorize a greater range of FLL types than most existing CEUS CAD systems, providing comparable accuracy to other ML techniques reported in the literature.

Secondly, this study expands our previous work [38] and advances the state-of-the-art in the field by proposing neural solutions that can perform an FLL diagnosis taking into account the CEUS video exploration. Taking into consideration the information provided by the video input data is a step forward in CEUS automatic diagnosis because it enables CAD to formulate an output based on spatio-temporal patterns, imitating the process performed by experienced practitioners, who not only observe separated frames (images and spatial patterns) but also the temporal characteristics of the entire exploration. For example, the “washout” feature describes a lesion becoming less bright (hypoenhancing) than the liver parenchyma, and its diagnostic significance depends on its timing.

In summary, the presented solutions offer (1) faster inference, making them appropriate in clinical workflows; (2) enhanced robustness across a range of imaging conditions; (3) less dependence on large, annotated datasets; and (4) accurate detection and classification of liver lesions.

This paper is structured as follows. Section 2 summarizes the state of the art. Section 3 introduces the materials and methods utilized throughout the study. Section 4 presents the experimental results using a deep neural architecture for CEUS images and video datasets. Finally, Section 5 concludes the paper.

## 2. State of the Art

### 2.1. Classical CNN-Based Approaches

Early applications of DL in CEUS and liver imaging have primarily relied on CNNs. Traditional computer vision and machine learning methods, such as multi-layer perceptrons radial basis function networks, and classical CNN architectures, were often used in place of hand-crafted features [39,40,41,42,43,44].

Both spatial and temporal information in CEUS examinations are clinically important: static contrast patterns are useful for diagnosing FLLs [45,46,47], while temporal dynamics extracted as time–intensity curves (TICs) provide complementary diagnostic clues [48,49].

For temporal modeling, CNNs have often been paired with recurrent architectures such as LSTM networks to capture enhancement dynamics across frames [50,51]. These CNN-based pipelines demonstrated strong performance but were limited by their local receptive fields and reliance on manual feature engineering in earlier stages.

### 2.2. Vision Transformers (ViTs)

With the success of the Transformer architecture in NLP, ViTs have been adapted for medical imaging. ViTs divide images into fixed-size patches and use multi-head self-attention to capture contextual relationships across the entire field of view [52,53,54,55,56,57,58].

This global attention mechanism makes them particularly suited for identifying fine-grained texture variations often present in liver lesions. Several studies [59,60,61,62] have reported ViTs surpassing CNNs in classification tasks when trained with sufficient data. In liver imaging, ViTs have been applied to tumor segmentation [63], microvascular invasion prediction (MVI-TR) [64], and cross-phase image fusion [65]. Adaptation strategies, such as AdaptFormer [66], aim to make ViTs more efficient for smaller medical datasets.

### 2.3. Hybrid CNN–Transformer Models

To overcome the limitations of pure CNN or pure Transformer designs, hybrid models have been proposed. These architectures leverage CNNs for efficient local feature extraction while employing Transformers to capture long-range dependencies [67,68,69,70,71]. Examples include TransLiver [65], which integrates convolutional encoders with Transformer blocks for multi-phase CEUS fusion, and CSWin [72], which introduces cross-shaped window self-attention for faster inference. Variants such as Swin Transformer, PVT, TNT, and DeiT [73,74,75,76] have also been designed with efficiency in mind, making them more suitable for clinical deployment. Hybrid methods extend further into generative paradigms, where Transformers are paired with Generative Adversarial Networks (GANs) to improve segmentation accuracy [77].

### 2.4. Video-Based Approaches

Because CEUS inherently provides dynamic video loops, video-based DL models have been investigated to jointly model spatial and temporal aspects. CNN–LSTM combinations [50,51,78], as well as 3D CNNs and Transformer-based video models, have shown promise in capturing both enhancement patterns and lesion morphology. These models remove the need for manual TIC extraction and better reflect the full diagnostic process used by radiologists. Video Transformers, in particular, extend ViTs to spatio-temporal contexts and are increasingly seen as a promising direction for CEUS analysis.

## 3. Materials and Methods

### 3.1. Materials

#### 3.1.1. Image Data

**Figure 1:**
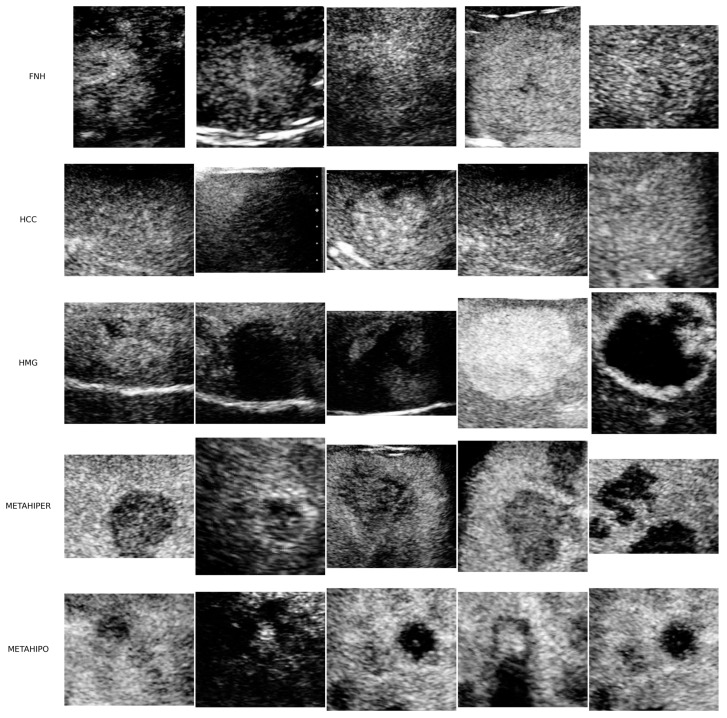
A total of 25 randomly selected ROI samples of different sizes from the UMFVBT CEUS image dataset. There are five pictures from each of the five available lesion types: FNH, HCC, HMG, Hypervascular Metastase (METAHIPER), and Hypovascular Metastase (METAHIPO) [80].

For the first part of this study, the data input is represented just by CEUS images, and the utilized data came from the gastroenterology and hepatology department of the “Victor Babes” University of Medicine and Pharmacy of Timisoara (UMFVBT) dataset. Ultrasound equipment Siemens Acuson S2000 was used for all the examinations in Timisoara, Romania. The UMFVBT dataset includes a total of 273 CEUS video files that were processed, featuring five types of FLLs. The dataset includes videos from 91 patients, with 16 cases of focal nodular hyperplasia (FNH), 30 cases of HCC, 23 cases of Hemangioma (HMG), and 11 cases each of Hyperplasia (HYPERM) and Hypoplasia (HYPOM). This distribution highlights the diversity of lesions within the cohort and offers valuable examples for model training and evaluation. A total of 12,119 pictures were captured during the process. These ROI images come in different sizes [79] as depicted in Figure 1.

To prevent echogenic gas bubbles from the injected agent that is required for this kind of procedure from disappearing, the ultrasound device’s probe does not remain in constant contact with the patient during the study. In total, 273 videos were analyzed, covering five different types of liver lesions (FNH, HCC, HMG, METAHIPER, and METAHIPO). Each group has a varied number of patients (FNH, 16 patients; HCC, 30; HMG, 23; METAHIPER, 11; METAHIPO, 11; total: 91).

ROIs have been manually defined by experienced physicians. Based on the Romanian Society of Ultrasound in Medicine and Biology categorization, all exams were performed by skilled operators (Level II = advanced and III = expert). The 2012 CEUS guideline procedure from the European Federation of Societies for Ultrasound in Medicine and Biology (EFSUMB) was adhered to in every examination. The authors of [11] state that histology, contrast-enhanced CT, and MRI were available to corroborate the final diagnosis.

The primary limitation of the image-based detection approach lies in the fact that although the training and testing sets consist of distinct images, these images may still originate from the same patient. This overlap can lead to an overly simplified evaluation process, which may not accurately reflect the performance of CAD in real-world patient scenarios.

#### 3.1.2. Video Data

**Figure 2:**
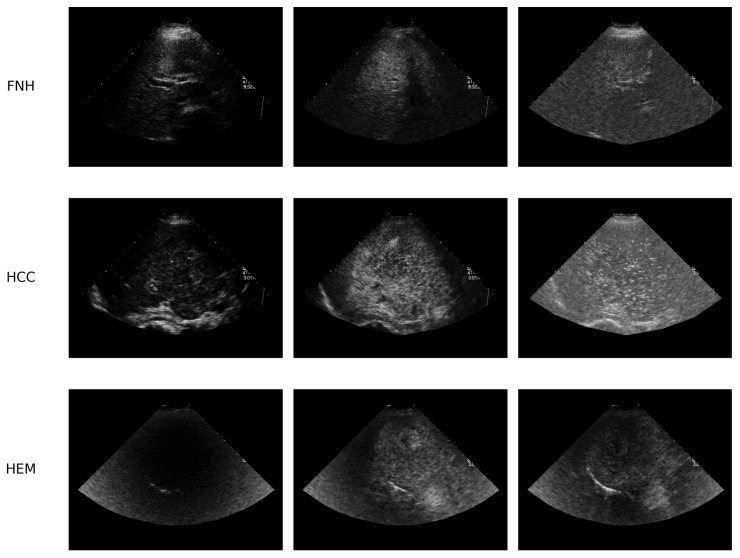
Randomly selected frames from the SYSU CEUS video dataset illustrating the three possible FLLs: FNH, HCC, and HEM.

For the second part of our study, the dataset includes dynamic video sequences acquired during clinical diagnostic procedures that covered a variety of pathological disorders, including hepatic lesions, e.g., HCC, HEM (HMG), and FNH. The SYSU-CEUS dataset includes 353 CEUS video sequences from three different types of FLLs (186 HCC, 109 HEM, and 58 FNH)—see Figure 2. The dataset, obtained at Sun Yat-sen University’s First Affiliated Hospital using an Aplio SSA-770A echograph from Toshiba Medical System, contains 186 malignant and 167 benign lesions. Videos with a resolution of 768 × 576 pixels were collected from different patients with large variations in the appearance and enhancement patterns (size, contrast, shape, and location) of focal lesions [81].

### 3.2. Methods

In this section, the deep neural network (DNN) architectures utilized in the current research are described as the design of a deep neural model represents a crucial aspect in CAD development, impacting its efficiency and performance, among other factors.

#### 3.2.1. DNN Architecture for CEUS Image Analysis

**Figure 3:**
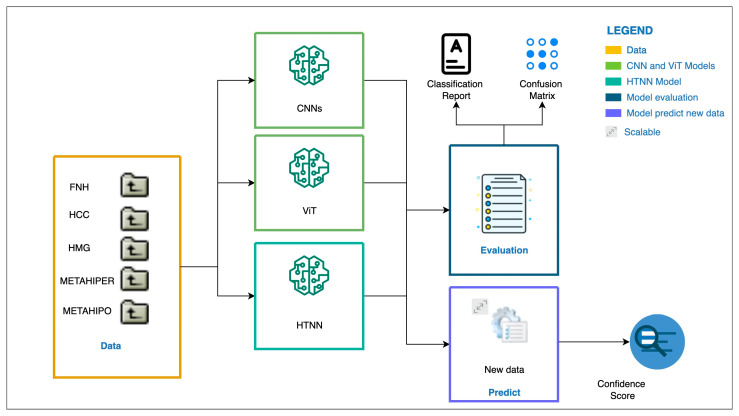
The experimental setup for the static CEUS image input data type [38] where we have newly introduced the HTNN architecture.

**Figure 4:**
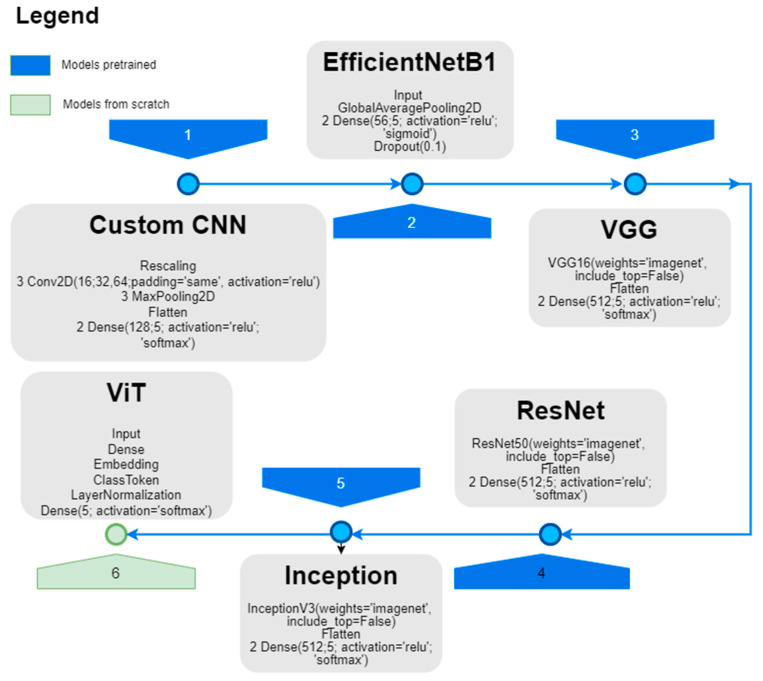
The experimental setup for CEUS images as input data, where we have newly introduced the HTNN architecture, including CNN models.

We designed an HTNN architecture (Figure 3) for the experimental setup and tested it against previously published CNN designs, such as a custom CNN, EfficientNetB1, VGG, ResNet50, Inception, and a previous design of a ViT model; the findings are presented in [47,80] and shown in Figure 4.

The workflow shown in the diagram above begins with the available labeled (FNH, HCC, HMG, METAHIPER, and METAHIPO) dataset—see the Data block of the diagram. The data were then fed into two types of models for processing: CNN models and ViT models. The CNN models include designs known to be suitable for image classification tasks, including a custom CNN, EfficientNetB1, VGG, ResNet50, and Inception. In addition, the ViT and HTNN models are utilized as (better) alternatives to the CNN models. Figure 4 depicts the tested architectures.

The neural models’ outputs are integrated into an Evaluation module. This module produces two key performance outputs: a Classification Report, which summarizes performance metrics such as accuracy, precision, recall, and F1-score, and a Confusion Matrix, which provides a detailed breakdown of model predictions versus actual labels which are very useful in evaluating CAD classification performance. In addition to assessment, trained models can be used to predict outcomes based on fresh, previously unknown data. The prediction results contain a confidence score to assess the accuracy of each prediction, which is a crucial aspect for clinical decision assistance.

**Figure 5:**
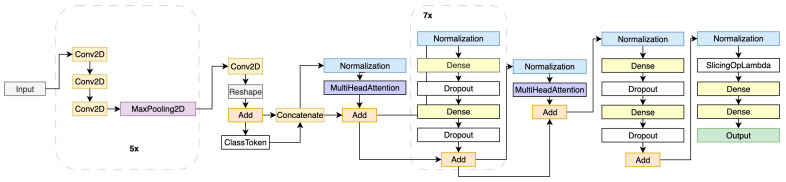
The proposed HTNN architecture for processing CEUS images [38].

The hybrid DL architecture shown in Figure 5 is intended to categorize the five liver lesions (FNH, HCC, HMG, METAHIPER, and METAHIPO) from the image data. Multiple layers of convolution (Conv2D + Activation) are applied directly to the input data (ROIs containing the lesions). The max pooling layer is then used to form a more compact feature representation. Before the data goes through the concatenation, normalization, and multi-head attention layers, a class token is inserted in order to capture intricate relationships. To improve learning, many Transformer blocks—such as layer normalization, dense layers, dropout, and multi-head attention—are added. The model then produces predictions for every type of lesion using both local and global characteristics, combining the best characteristics of the CNN and Transformer. Using a CNN as a frontend improves sample efficiency and training stability by extracting low and mid-level local features, being most impactful in smaller datasets. The Vision Transformer layer performs global modeling, capturing long-range dependencies and context; this feature is the most useful when dealing with large-scale datasets. This hybrid interaction carefully mixes local CNN biases with global Transformer reasoning, thus leading to big performance gain.

#### 3.2.2. DNN Architecture for CEUS Video Analysis

Our next proposal for this study is to use an end-to-end DNN-based solution to classify the focal lesion type. This approach will avoid the necessity of some hand-crafted feature engineering tasks, i.e., ROI selection, as DL architectures are capable of extracting meaningful features directly from the raw input video data. For this task, two different DNN architectures capable of directly processing video input data were used. Moreover, these recurrent DNN architectures can capture temporal features, a very important aspect in the context of a CEUS-based diagnosis process.

**Figure 6:**
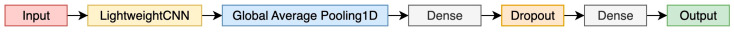
The proposed lightweight CNN architecture for CEUS video data.

Firstly, we designed a custom, lightweight, frame-wise CNN for video classification—see Figure 6. This model is specifically designed to extract spatial features from individual frames while effectively aggregating these features over time. Each frame is processed using a compact MobileNetV2 backbone, which operates with a reduced width (alpha = 0.35) to maintain efficiency. This backbone is integrated within a TimeDistributed layer (32, 64, 64, 3) to ensure the sharing of weights across all frames. The temporal features obtained are summarized through GlobalAveragePooling1D (32, 1280), followed by a small fully connected layer that incorporates a dense rectified linear unit (ReLU) activation function (1280) [82], dropout (0.5) for regularization, and a softmax output layer (64) for classification purposes. The model is trained using the Adam optimizer in conjunction with a custom focal loss function that includes class weighting. This approach effectively addresses class imbalance by placing greater emphasis on instances that are more challenging to classify. The total number of parameters is ~492 k.

Secondly, as an additional baseline, we consider a compact depthwise separable CNN with a bidirectional LSTM (BiLSTM) [78] to capture both spatial and temporal dynamics in video sequences. Each frame passes through the SmallDepthwiseCNN block, which uses separable convolutions (32, 64, and 128 filters with 3 × 3 kernels) and pooling to efficiently extract hierarchical spatial features while reducing parameters. The frame-level features are then aggregated over time using a TimeDistributed wrapper, feeding into a bidirectional LSTM with 64 hidden units and dropout (0.3 recurrent + 0.3 standard) to learn temporal dependencies in both directions. The sequence-level representation is further refined with a fully connected layer of 64 ReLU units, followed by 50% dropout for regularization, and finally mapped to the target classes through a softmax output. Training uses the Adam optimizer with a learning rate of 10^−4^, a custom focal loss function, and accuracy as the evaluation metric, making it well-suited for multi-class sequence classification with relatively lightweight computation.

**Figure 7:**
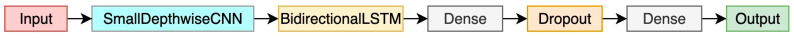
The proposed lightweight *CNN + Bidirectional LSTM* architecture for CEUS video data.

With a total parameter count of ~118 k, this model is lightweight and suitable for moderate-sized time-series tasks (Figure 7).

**Figure 8:**
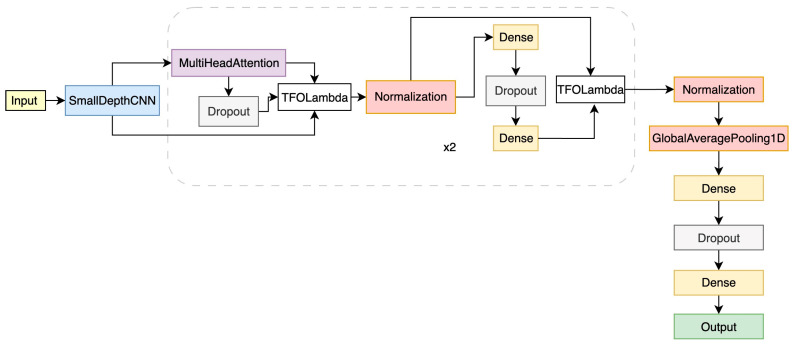
The proposed ViViT architecture to process CEUS video data.

Thirdly, the Video Visual Transformer (ViViT) model integrates a lightweight depthwise separable CNN with a Transformer encoder to capture both spatial and temporal dependencies in video data. Each frame first passes through the SmallDepthwiseCNN block, which applies separable convolutions with 32, 64, and 128 filters; batch normalization; ReLU activations; pooling layers; and a global average pooling step to extract compact frame-level features efficiently. These frame features are then processed sequentially by a stack of Transformer encoder blocks, where multi-head self-attention (head size = 32, 4 heads) and feed-forward layers of 128 units learn temporal relationships and contextual interactions across frames. Afterward, global average pooling across the temporal dimension condenses the sequence representation, followed by a dense ReLU layer of 64 units and 50% dropout for regularization (Figure 8). The final softmax layer outputs class probabilities, while training uses the Adam optimizer with a learning rate of 1 × 10^−4^ and a focal loss function (with tunable α and γ = 2.0) to address class imbalance and focus learning on harder examples. The total number of parameters is extremely low, ~219 k.

## 4. Experimental Results

### 4.1. Image-Based Evaluation

**Figure 9:**
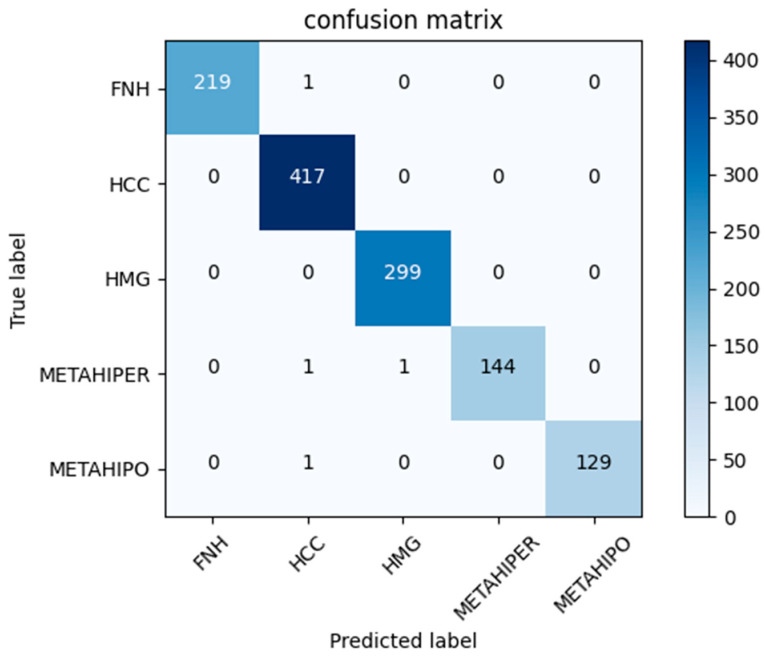
Confusion matrix for the HTNN model.

The HTNN architecture behavior for CEUS FLLs classification, in comparison with other state-of-the-art methods, is presented in this section (see Table 1, Table 2 and Table 3 and Figure 9). Comparing SOTA methods is challenging for several reasons, primarily due to the varying number of lesions and, more importantly, the way images are divided into training and test sets. In all approaches analyzed, except for [47], the patient origin of the images was not taken into account when making this division. This oversight helps to explain the high accuracy rates reported in Table 2. However, in clinical practice, a trained model will be tasked with providing a diagnosis for a patient whose imaging data were not included in the model’s training. That is why a Leave-One-Patient-Out evaluation procedure more accurately represents the model’s performance.

For our case, 12,119 ROI images were organized into five folders, corresponding to the five lesions. We used 1211 (10%), 1212 (10%), and 9695 (80%) images for testing, validation, and training, respectively. To obtain the input shape of (512, 512, 3), all the images were resized and shuffled without any augmentation.

The experiments were performed using the following setup:

- Hardware architecture: CPU: AMD RYZEN 7 5800X, RAM: 64 GB, GPU: NVIDIA GeForce RTX 3090, 24 GB RAM.
- Software framework: TensorFlow 2.11.1, Python 3.8.0, Ubuntu 20.04 LTS 64-bit.

For the training process, we implemented the following configuration: categorical cross-entropy loss, the Adam optimizer, and a learning rate of 0.0001.

The results show that ResNet50 and Inception achieved the highest validation accuracy scores (99.17% and 99.34%), though Inception had a much higher loss, suggesting less stable learning. VGG also performed very well (97.61% accuracy with low loss) but required the longest training time and a large number of parameters. CNN and EfficientNetB1 reached moderate accuracy (~92%) with relatively low training times, while ViT underperformed with the lowest accuracy (86.54%) despite having the fewest parameters. Overall, ResNet50 provided the best balance between high accuracy and low loss, but at the cost of high model complexity (Table 1).

Achieving high accuracy in our models is promising, but clinical adoption requires computational efficiency as well. Larger architectures, such as ResNet50 (74.9 million parameters) and Inception (38.6 million parameters), deliver accuracy rates of 99.17% and 99.34%, respectively. However, their memory demands and longer inference times may render them unsuitable for resource-constrained environments.

In contrast, lightweight models like CNN (5.1 million parameters) and EfficientNetB1 (6.6 million parameters) maintain competitive accuracy of around 92% while offering smaller memory footprints and faster inference times, making them better suited for portable or point-of-care devices.

As main hyperparameters, we used 64 patches, 32 batch sizes, a 1 × 10^−4^ learning rate, the Adam optimizer, categorical cross-entropy loss, 100 training epochs, 5 classes, 8 layers, 12 hidden dimensions, 768 MLP dimensions, 12 heads, and a dropout rate of 0.4.

### 4.2. Video-Based Evaluation

In the video CNN setup, the dataset loader function prepares video data for training by extracting fixed-length frame sequences, applying preprocessing, and splitting into training, validation, and test sets while preserving patient-level grouping. Each video is read frame by frame, resized to a target image size (64, 64), normalized to the [0, 1] range, and optionally augmented with flips, small rotations, and brightness jitter for specified classes (FNH and HEM) to improve generalization. Frames are sampled at a defined interval (frame_skip = 5) and grouped into sequences of consistent length (frames_per_sequence = 32) to serve as model inputs. Labels are mapped to integer indices, converted into one-hot encoded vectors, and kept aligned with patient identifiers to ensure subject-independent evaluation. Finally, stratified group-aware splitting using GroupShuffleSplit ensures that frames from the same patient appear in only one subset, producing train, validation, and test sets with balanced class representation and no data leakage. First, GroupShuffleSplit divides the entire dataset (186 HCC patients, 109 HEM patients, and 58 FNH patients) into a training set (70%) and a temporary set (30%), using patient IDs as grouping keys so that all sequences from the same patient are assigned to only one split. Next, the temporary set is further split evenly into validation (15%) and test (15%) subsets using another GroupShuffleSplit, again grouping by patient IDs to avoid overlap. This approach guarantees that the training, validation, and test sets are disjointed at the patient level, preventing data leakage and providing a fair evaluation of the model’s generalization to unseen patients.

We used augmentation data that introduces variability into training frames to improve model generalization by applying random spatial and brightness transformations. With a 50% chance, the frame is flipped horizontally to simulate mirrored perspectives. Next, a random rotation between −15° and +15° is applied around the frame’s center using an affine transformation, mimicking slight camera angle changes. Finally, brightness jitter is introduced by multiplying pixel values with a random factor between 0.8 (darker) and 1.2 (brighter), with values clipped to the valid [0, 1] range. Together, these augmentations help the model become more robust to real-world variations in orientation and lighting conditions.

The callbacks are used to improve training stability and model performance by dynamically managing checkpoints, early stopping, and learning rates. The ModelCheckpoint callback saves only the model weights, keeping the best version based on maximum validation accuracy. The EarlyStopping callback stops training if validation accuracy does not improve for 30 consecutive epochs, restoring the best weights to prevent overfitting and wasted computation. Finally, the ReduceLROnPlateau callback monitors validation loss and reduces the learning rate by half whenever progress stalls for 5 epochs, with a minimum limit of 1 × 10^−6^, enabling finer convergence and helping the optimizer escape plateaus, training for 100 epochs with a batch size of 4, using ReLU and Softmax activation functions [82].

In the depthwise separable CNN model integrated with a bidirectional LSTM architecture, we ensured that the conditions for data preprocessing and evaluation settings were consistent with the video CNN setup.

In this Transformer setup, we integrated a small CNN model with a Transformer model that utilizes specifically tailored focal loss parameters and robust training strategies. To enhance generalization, data augmentation is selectively applied to the FNH and HEM classes. The model’s input shape is derived from the preprocessed training sequences, while the number of classes is based on one-hot encoded labels. We employ a custom focal loss with γ = 2.0 and class-specific weights of [1.4, 0.5, 1.0]. This approach emphasizes the FNH and HEM classes while down-weighting the more prevalent HCC class to improve precision in our predictions. The Transformer encoder comprises two blocks, each utilizing multi-head attention with four heads (head size = 32) and feed-forward layers containing 128 units. These elements are stacked following a lightweight CNN feature extractor. Training is conducted over a maximum of 100 epochs with a batch size of 4. We implement the ModelCheckpoint functionality to save the best weights based on validation accuracy. EarlyStopping is utilized to stop training after 30 epochs of stagnation, and ReduceLROnPlateau is applied to halve the learning rate when the validation loss plateaus, ensuring stable convergence throughout the training process.

Table 4 presents a comparative overview of classification performance across multiple architectures, highlighting trade-offs in handling three classes: FNH, HCC, and HEM.

The lightweight CNN achieves solid overall accuracy (~0.88) and perfect classification of HCC but struggles with FNH (low precision) and shows only moderate performance on HEM. This suggests a bias in favor of the dominant class.

By contrast, hybrid models such as CNN + BiLSTM (~0.75) and ViViT (~0.88) improve balance by capturing temporal dependencies and contextual relationships. These methods yield higher macro-averaged precision, recall, and F1-scores, reducing misclassification of FNH and HEM compared to the baseline CNN. Recurrent and attention-based mechanisms therefore provide more robust, equitable performance across all categories, especially for underrepresented or difficult cases.

**Figure 10:**
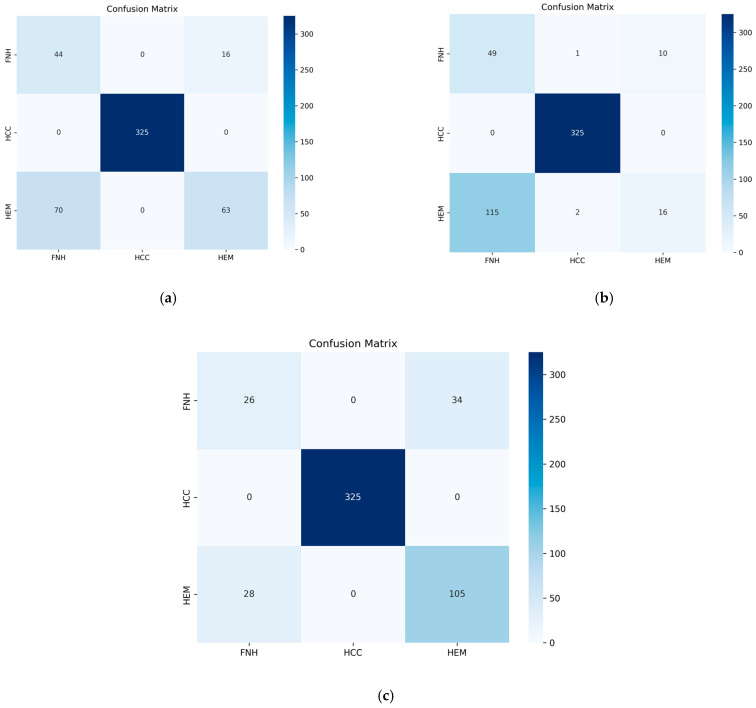
Confusion matrix on the test set. (**a**) Video lightweight CNN model; (**b**) video CNN + LSTM model; (**c**) ViViT model.

The confusion matrices provide insight into how various architectures approach class separation within the test set—see Figure 10. All three models—(a) the lightweight CNN, (b) CNN + BiLSTM, and (c) ViViT—successfully achieve perfect classification for HCC, with 325 correct predictions and no errors, demonstrating the clear distinguishability of this class.

However, their performance in differentiating between FNH and HEM varies considerably. The lightweight CNN (a) correctly identifies 44 FNH cases and 63 HEM cases but shows substantial confusion, with 70 HEM instances misclassified as FNH and 16 FNH instances misclassified as HEM. The CNN + BiLSTM model (b) correctly classifies 49 FNH and only 16 HEM cases, while a large proportion of HEM cases (115) are misclassified as FNH, indicating poor separation of these two classes.

The ViViT (c) demonstrates the most balanced performance, accurately identifying 26 FNH and 105 HEM cases. Although some confusion between FNH and HEM remains (34 FNH predicted as HEM and 28 HEM predicted as FNH), this model achieves the best trade-off between the two.

In conclusion, while HCC is consistently identified with high accuracy across all models, the ViViT exhibits superior generalization capabilities when addressing the minority classes (FNH and HEM). This highlights the effectiveness of attention mechanisms in capturing subtle inter-class differences that conventional CNN-based architectures struggle with.

**Figure 11:**
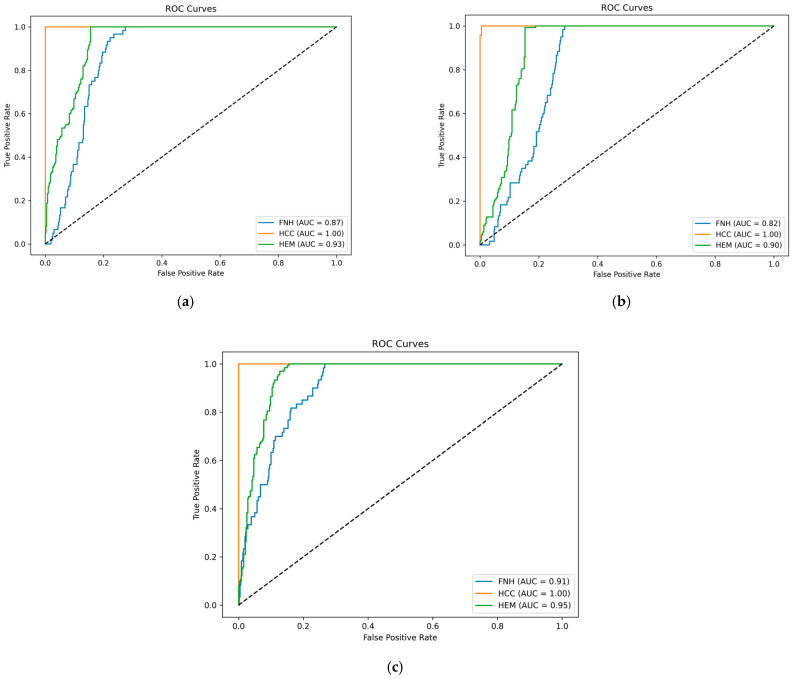
ROC curves on the test set. (**a**) Video lightweight CNN model; (**b**) video CNN + BiLSTM model; (**c**) ViViT model.

Figure 11 illustrates the ROC curves for three models evaluated on the test set: (a) a lightweight video CNN, (b) a video CNN integrated with BiLSTM, and (c) a ViViT. All three models exhibit exceptional performance in classifying HCC, consistently achieving an area under the curve (AUC) of 1.00.

For the remaining classes, performance varies. The lightweight CNN (a) demonstrates solid results, with AUCs of 0.87 for FNH and 0.93 for HEM. The CNN + BiLSTM model (b) shows slightly lower performance, achieving AUCs of 0.82 for FNH and 0.90 for HEM, suggesting that the addition of temporal modeling did not yield significant improvements in distinguishing between these classes.

In contrast, the ViViT (c) achieves the strongest overall performance, with the highest AUCs of 0.91 for FNH and 0.95 for HEM. This highlights its superior generalization capability and effectiveness in capturing subtle inter-class differences in multi-class video-based classification. In conclusion, while HCC is easily and perfectly separable across all models, the ViViT provides the most robust and balanced performance when addressing the more challenging distinction between FNH and HEM.

To optimize model training on a small and imbalanced video dataset, we selected a batch size of 4 and a learning rate of 1 × 10^−4^, along with relatively high dropout rates of 0.5 in the fully connected head and 0.3 in the Transformer/LSTM layers. This configuration balances hardware constraints, training stability, and regularization.

A small batch size fits GPU memory for long frame sequences and enhances generalization on limited data. The low learning rate supports stable weight updates, which is crucial for complex architectures like attention mechanisms and LSTMs. The strong dropout rates help mitigate overfitting, while four attention heads allow for the effective capture of temporal relationships without excessive computational requirements.

## 5. Conclusions and Future Work

The study presents a comprehensive analysis of DNN-based liver lesion classification methods, using images or video sequences obtained from CEUS exploration as input.

As elements of novelty, we proposed, for the case of image data input, a hybrid architecture that effectively combines CNN layers (to capture local spatial features) with Transformers that manage global context, thereby enhancing the differentiation of lesions, resulting in a slim Visual Transformer model called HTNN. The resulting architecture can address common medical dataset issues, such as small sample sizes. Compared to one of our previous works [80], we successfully streamlined the design by reducing the number of parameters from 57.6 million to 8.6 million. This optimization led to a notable reduction in training time to approximately half of the time previously required. Comparing our findings with those of the latest methodologies is challenging due to differences in datasets, experimental setups, and evaluation techniques. However, our ViT design for image tasks performs better than other approaches in terms of classification accuracy and the number of classes/FLLs. Medical imaging problems with limited information can benefit from the hybrid HTNN model’s ability to efficiently balance model complexity and performance. The potential for implementation in real medical scenarios, where computational resources and annotated data are frequently scarce, is highlighted by the ability to reduce model parameters without affecting accuracy.

The next contribution of our work goes into the diagnosis of FLLs from CEUS video data input. We firstly introduce a lightweight video 3D CNN architecture tailored for the analysis of medical video data for focal liver lesion classification. The second proposal presents a combination of a lightweight video 3D CNN with LSTM networks, yielding competitive results in the field. Our third proposal explores a lightweight hybrid Video Visual Transformer, called ViViT, which addresses the current gap in research regarding video detection, as most studies predominantly focus on image data for the identification of liver tumors. These setups demonstrated significant improvements in performance relative to prior studies [78].

Models use full or sampled video frames to implicitly identify lesion clues through video-level supervision, removing the need for manual regions of interest (ROIs) during inference. Video models have outperformed image-based baselines and have matched or exceeded some human readers in certain metrics [89].

Future initiatives will focus on evaluating image and video models in a range of clinical contexts, including the incorporation of additional CEUS datasets and expanding DL-based diagnostics to US imagery. Many datasets remain inaccessible due to privacy concerns.

Additionally, it is essential to benchmark inference times and memory usage across various hardware configurations to balance diagnostic accuracy with real-time clinical usability.

A future direction of our research involves the use of pre-trained models from Hugging Face, specifically a comparative analysis of three additional Transformer-based models. Subsequent studies will expand this comparison to include more models.

A possibility to elevate these concerns is given by a relatively new learning paradigm called Federated Learning (FL), a decentralized learning technique where multiple devices (clients) collaboratively train a model without sharing their raw data. Each device trains a local model on its own data, and then only the model updates (not the data) are sent to a central server. This learning paradigm offers some key advantages, e.g., those related to the privacy-preserving aspect (raw data never leave the device), distributed training (it happens across many devices), and efficiency (reduces the need for centralized data collection) [90].

Additionally, we will examine various validation methodologies to strengthen the generalization and robustness of these models. One potential approach for improvement involves addressing the unequal distribution of data across the classes to enhance overall performance. Additionally, we will aim to fine-tune the parameters and conduct a comparative analysis with the baseline models.

Testing alternative, more complex Transformer models is a valuable future approach to obtain better results, although their size and hunger for data might represent important hindrances. Options like Transformer-XL [91], Swin Transformer [74], or Aggregating Nested Transformers and Cross-Attention [92] can offer substantial advantages through efficiency-enhancing architectures, improved long-term relationships, or computational cost optimization. Therefore, investigating these models and contrasting them with the baseline Transformer may offer valuable insights for enhancing performance in the task under study. We will also consider pre-training models on general action recognition datasets such as Kinetics-400 before fine-tuning them on domain-specific medical datasets that include organs other than the liver (for example, the kidney, spleen, or multi-organ segmentation using datasets such as BTCV or LiTS [93] for CT/MR images). This method can increase model resilience and generalization, particularly when medical datasets are small or diverse [94,95].

Furthermore, we intend to investigate improved MLOps strategies to enable rapid model deployment, real-time inference, and effective lifecycle management in clinical contexts. We plan to add multimodal data sources and expand the dataset with larger patient cohorts to enhance classification accuracy and clinical value, ultimately increasing the model’s potential for general use in medical imaging diagnostics.

## Figures and Tables

**Table 1:** Comparison of architectures used.

Model	Validation			
	Accuracy (Max)	Loss (Min)	Total Params	Total Training Time [min]
CNN	91.99%	0.3274	5,144,357	8
EfficientNetB1	91.82%	0.5992	6,647,253	8
VGG	97.61%	0.1171	24,154,949	53
ResNet50	99.17%	0.0415	74,971,013	51
Inception	99.34%	1.2361	38,583,077	34
ViT	86.54%	0.6431	2,584,517	17

**Table 2:** Comparison of state-of-the-art methods.

Ref.	Lesions	General Accuracy [%]
Hassan et al. [83]	Cyst, HEM, HCC	97.2
Pan et al. [84]	FNH, HCC	93.1
Guo et al. [85]	Malign, Benign	90.4
Vancea et al. [86]	HCC	80.3
Wu et al. [87]	HCC, CH, META, LFS	86.3
Streba et al. [88]	HCC, HYPERM, HYPOM, HEM, FFC	87.1
Căleanu et al. [47]	FNH, HCC, HMG, HYPERM HYPOM	88.0
Mercioni et al. [80]	FNH, HCC, HMG, METAHIPER, METAHIPO	97.5
HTNN (ours)	FNH, HCC, HMG, METAHIPER, METAHIPO	97.77

**Table 3:** Classification report for the HTNN model in five FLLs: FNH, HCC, HMG, Hypervascular Metastase (METAHIPER), and Hypovascular Metastase (METAHIPO).

	Precision [%]	Recall [%]	F1-Score [%]	Samples
FNH	100	100	100	222
HCC	99	100	99	394
HMG	99	99	99	324
METAHIPER	99	99	99	134
METAHIPO	100	99	99	138
Accuracy			99	1212
Macro avg	100	99	99	1212
Weighted avg	99	99	99	1212

**Table 4:** Classification report for the proposed neural video processing architectures.

Architecture	Lesion Type	Precision	Recall	F1-Score	Samples
Video lightweight CNN	FNH	0.39	0.73	0.51	60
	HCC	1.00	1.00	1.00	325
	HEM (HMG)	0.80	0.47	0.59	133
	macro avg	0.73	0.74	0.70	518
	weighted avg	0.88	0.83	0.84	518
	accuracy		0.83		518
Video CNN + LSTM	FNH	0.30	0.82	0.44	60
	HCC	0.99	1.00	1.00	325
	HEM (HMG)	0.62	0.12	0.20	133
	macro avg	0.64	0.65	0.54	518
	weighted avg	0.81	0.75	0.73	518
	accuracy		0.75		518
ViVit	FNH	0.48	0.43	0.46	60
	HCC	1.00	1.00	1.00	325
	HEM (HMG)	0.76	0.79	0.77	133
	macro avg	0.75	0.74	0.74	518
	weighted avg	0.88	0.88	0.88	518
	accuracy		0.88		518

## Data Availability

The CEUS SYSU video dataset is freely available, see [81,96]. The UMFVBT CEUS data are available upon request from the corresponding author of [11] and it is not publicly available.
